# Flow and Heat Transfer in the Tree-Like Branching Microchannel with/without Dimples

**DOI:** 10.3390/e20050379

**Published:** 2018-05-18

**Authors:** Linqi Shui, Jianhui Sun, Feng Gao, Chunyan Zhang

**Affiliations:** 1 Key Laboratory of NC Machine Tools and Integrated Manufacturing Equipment of the Education Ministry & Key Laboratory of Manufacturing Equipment of Shaanxi Province, Xi’an University of Technology, Xi’an 710048, China; 2 Xi’an Aerospace Composite Materials Research Institute, Xi’an 710025, China

**Keywords:** gas turbine cooling, tree-like branching microchannel, dimple, heat transfer enhancement, friction factor

## Abstract

This work displays a numerical and experimental investigation on the flow and heat transfer in tree-like branching microchannels and studies the effects of dimples on the heat transfer enhancement. The numerical approach is certified by a smooth branching microchannel experiment. The verification result shows that the SSG turbulence model can provide a reasonable prediction. Thus, further research on the convective heat transfer in dimpled branching microchannels is conducted with the SSG turbulence model. The results indicate that the dimples can significantly improve the averaged heat transfer performance of branching microchannels, and the heat transfer increment of the branch segment increases with the increase in the branching level. However, the flow dead zones in some dimples at bifurcations and bends suppress the turbulent flow and heat transfer. Furthermore, the *Nu* number ratio (*Nu*_a_/*Nu*_s_) and thermal enhancement factor (*η*) both monotonously decrease as the *Re* number increases, while the friction factor ratio (*f*_a_/*f*_s_) changes nonlinearly. The entropy generation rates of ${\dot{S}}_{t}$ and ${\dot{S}}_{p}$ in all dimpled cases are lower than those in the smooth case, and the dimpled case with the streamwise spacing to diameter ratio *s*/*D* = 3 obtains the lowest value of augmentation entropy generation (*N_s_*) under the high *Re* number conditions. *Nu*_a_/*Nu*_s_, *f*_a_/*f*_s_, and *η* decline with the increase in the streamwise spacing to diameter ratio (*s*/*D*) from 3 to 9; therefore, the dimpled case with *s*/*D* = 3 shows the best overall thermal performance.

## 1. Introduction

The modern gas turbine is the core of advanced energy power systems. Its thermal efficiency and power output increase as gas inlet temperature rises. Generally, the hot gas temperature is far more beyond the melting point of the blade material. As a result, the blades need to be cooled by an efficient and reliable cooling system [1]. Currently, the research interests regarding cooling technology mainly include the optimization of cooling structure parameters, the improvement of complex cooling strategies, and the development of new cooling technologies based on innovation cooling theories. For blade internal cooling, the dimple cooling technology and the microchannel cooling technology both show remarkable cooling performance.

Dimples employed on surfaces can induce the self-organized vortex motions and facilitate turbulent mixing in heat transfer. To investigate the thermal performance affecting factors, a number of studies have been implemented. Burgess et al. [2] and Ekkad et al. [3] found that, in the turbulent flow conditions, the heat transfer coefficient and flow friction factor are both decreased with the reduction of the channel height but increased with rises in the dimple depth. In a study by Moon et al. [4], it was pointed out that the channel height has little influence on the heat transfer coefficient and flow friction factor in the relative height range of 0.37–1.49. The effects of dimple shapes, surface curvatures, and dimple spaces on heat transfer and hydrodynamics in the dimpled channel or plate have also been investigated [5,6,7].

Schukin et al. [8] introduced the dimpled structure into heat transfer augmentation for gas turbine blade cooling. Ligrani and Mahmood [9,10], Murata et al. [11], Xie et al. [12], and Shen et al. [13] severally studied the heat transfer and flow characteristics in straight or serpentine channels with dimpled walls. The results showed that the average heat transfer enhancement of dimpled channels can be 2–3 times the turbulent flow in a smooth channel without significant increasing the flow friction factor. Additionally, compared to the ribs, the dimples have the same level of heat transfer performance but only half the pressure loss.

With the high demand for the gas turbine blade cooling system, the dimple cooling technology is often combined flexibly with other cooling technologies to obtain higher cooling effectiveness. Kanokjaruvijit and Martinez-Botas [14] experimentally investigated the heat transfer performance and pressure distribution of an inline array of jets impinging on an array of dimples and the influence factors of the coupling effects of impingement and dimpled channel flow. Shen et al. [15] experimentally and numerically studied the heat transfer and pressure drop characteristics in blade trailing edge trapezoidal and rectangular cooling passages with dimples under the effect of ejection slots. Lauffer et al. [16] set up a rectangular dimpled channel for gas turbine hot components and designed different configurations of rib promoters in the corner regions to heat transfer enhancement. Chang et al. [17] reported on heat transfer and pressure drop features of radially rotating ribbed channels with and without dimples. These above results show that the blade composite cooling structures with dimples can provide excellent cooling effectiveness without large pressure loss penalty.

In addition, dimple cooling technology, the new concept of microchannel cooling technology for gas turbine blades has been proposed [18]. Highly distributed microchannels not only have a high local wall heat transfer coefficient but also a large convection surface area per unit volume. In addition, learning from the most efficient energy and mass transport systems in the natural world, such as the root system of plants as well as mammalian circulatory and respiratory systems, in order to design tree-like branching network microchannels as blade internal cooling structures, can provide the enough cooling efficiency and particularly reduce the flow pressure loss as well as improve the cooling uniformity, consequently alleviating the thermal loads of the blade wall.

Inspired by natural systems, Bejan [19] first put forward the constructal theory to direct the microchannel networks design for an efficient heat transfer process. By employing an optimization approach to minimize flow loss at minimal volume, West et al. [20] deduced that the ratio of diameters and lengths between the consecutive bifurcation levels can be represented by *d_k_*_+1_/*d_k_* = *n*^−1/Δ^ and *l_k_*_+1_/*l_k_* = *n*^−1/Δ^, respectively, where *n* denotes the number of bifurcations at each tube. Pence [21] designed a fractal-like bifurcating network and then predicted its pressure drop and heat transfer coefficient distribution. Results showed the robustness of branching channels as compared to traditional parallel straight channels. A comparison of the thermal and hydraulic characteristics between bifurcating channels and serpentine channels was conducted by Senn and Poulikakos [22]. Results revealed that the bifurcating channel possesses a stronger thermal exchange ability but costs only half the pressure loss. Chen and Cheng [23] developed an H-shaped bifurcating channel network for cooling chips. In the study, it was found that the fractal bifurcating channel has a lower pumping power requirement, better cooling effectiveness, and more uniform temperature distribution compared to the serpentine channel.

To obtain the optimum structure for cooling chips, parametric studies on the influences of blockage, the number of levels, branches, and tubes on the flow and heat transfer characteristics of branching network channels were conducted by Wang et al. [24]. Wechsatol et al. [25] optimized the channel length distribution in a laminar and fully developed flow condition for the disk-shaped geometry. Chen et al. [26] carried out the constructal optimizations of heat exchangers and found that the X-shaped heat exchanger has a greater thermal efficiency than the H-shaped one. Substituting the traditional helical cooling channels network, Xia et al. [27] designed a novel tree-like branching channels network in a high-speed spindle’s cooling sleeve to boost the spindle’s overall convective cooling performance. By changing the branching angle, Chen et al. [28] researched the influence of geometrical parameters on the pressure drop and mixing efficiency for Y-shaped and T-shaped micromixers.

As mentioned above, promising cooling technology that involves the adoption of tree-like branching microchannels has been widely applied in many engineering fields, but studies related to the area of advanced blade cooling technology are less reported. Bunker [18] introduced various macro and micro cooling techniques for gas turbine hot components and indicated that microchannel cooling can gain significant performance, for instant cooling flow reductions of 40% and thermal stress reductions approaching 50%. In the study of Sun et al. [29], they observed that the bifurcating channels display good effects on the balancing cooling performance with pumping power loss. Devore and Kaufman [30] disclosed a bifurcating blade core cooling arrangement to limit the number of inlets to the channel and controlling pressure loss near the film holes. Ahmad et al. [31], who are from Siemens AG, reported a cooling arrangement of bifurcating passages that paralleled a leading edge and a trailing edge of the airfoil in order to maintain cooling performance in the event of damage to the airfoil and consequently to a cooling passage. A detailed numerical and experimental study on the complex flow and convection heat transfer characteristics of tree-like bifurcating microchannels for blade cooling was recently conducted by Shui et al. [32,33,34]. Results showed that the tree-like branching microchannel provides a desirable low flow friction factor and good cooling effectiveness under a relatively high *Re* number condition.

Although previous research works have respectively demonstrated the heat transfer and flow friction characteristics of dimple cooling and tree-like branching microchannels cooling, the detailed structure of the flow field and mechanism of heat transfer enhancement in the new structure of the tree-like branching microchannels combined with dimples have never been revealed. The present work focuses on the heat transfer augmentation of the complex flow in a branching microchannel with arrays of dimples. In order to better compare the thermal performance of the dimpled branching microchannel with that of the smooth branching microchannel, an air cooling experiment and numerical simulations in the corresponding smooth case were conducted. Furthermore, numerical simulations for three dimple arrangement patterns were performed, and the *Nu* number ratio, the friction factor ratio, the thermal enhancement factor, the entropy generation rate, and the augmentation entropy generation number in the dimpled branching microchannels were investigated.

## 2. Experimental System and Data Reduction

### 2.1. Experimental Setup

**Figure 1:**
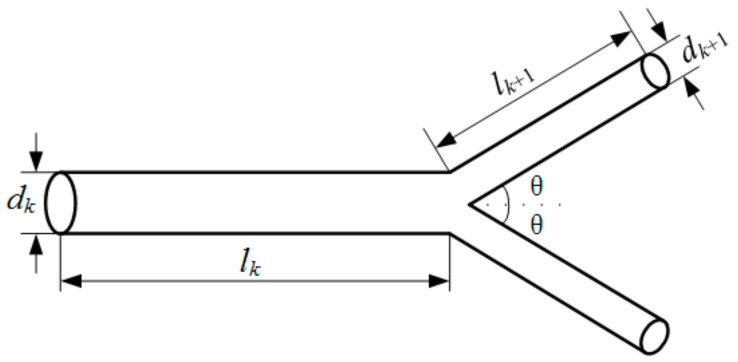
The geometric parameters of the tree-like branching microchannel.

**Figure 2:**
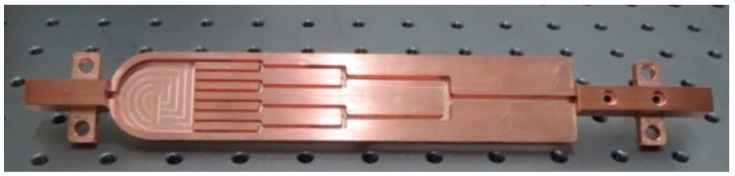
A photo of the cooper substrate.

A tree-like bifurcating network possesses self-similar characteristics, namely, a new branch can be generated on the basis of similar rules. As demonstrated in Figure 1, considering its fabricability, each tube was divided into two branches at the next level, and the bifurcating angle was *θ* = 90°. Thus, every lower-order tube continuously bifurcated out more tube. Based on the suggestion made by West et al. [20] for channel flow networks with few bifurcations, the branching channel network in the blade adopted the following branching ratios:

(1) $$d_{k+1}/d_{k}=n^{-1/2}$$

(2) $$l_{k+1}/l_{k}=n^{-1/2}$$

where *d* denotes the hydraulic diameter, and *l* denotes the length of a channel segment. For the present study, *n* = 2. Subscript *k* represents the lower-order bifurcating level and subscript *k* + 1 represents the higher-order bifurcating level at a branch. The first bifurcation emanating from the entrance plenum was the 0th-order branch, namely, *k* = 0. The dimensions of the rectangular flow network analyzed are listed in Table 1. Here, *H* denotes the channel height, *W* denotes the channel width, and *S* denotes the transverse channel length. The test section was made of T2 copper, including a 1-mm-thick cover plate and a 5-mm-thick substrate. The plate of the cover and the substrate were sealed by tin soldering. The branching network microchannels with a 3 mm height are micro milled out. Figure 2 presents the photo of the microchannels in the substrate.

**Figure 3:**
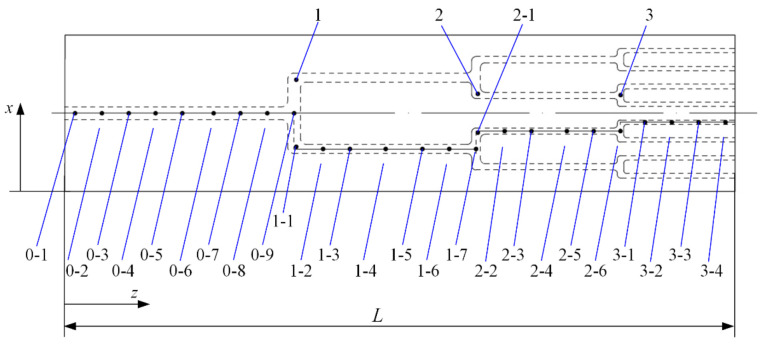
The layout of thermocouples measuring points.

**Figure 4:**
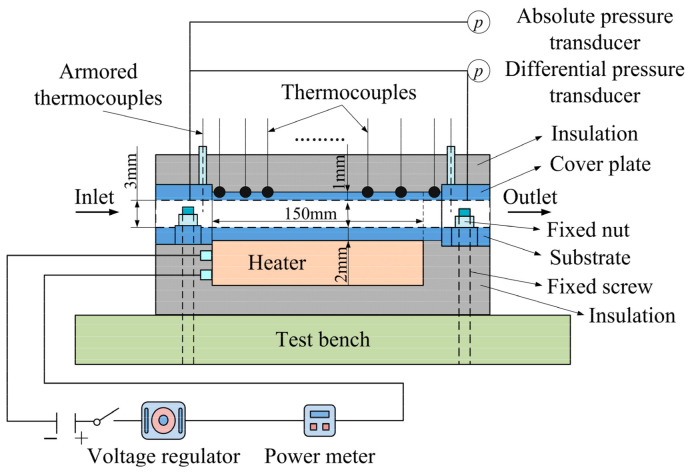
Design of the test section.

To measure the streamwise wall temperature of the branching network microchannels, 29 calibrated type-J thermocouples were glued at the exterior surface of the cover plate, and the thermocouples locations are shown in Figure 3. Eight, five, four, and four thermocouples were respectively decorated along the projecting centerline of each branch segment from Bifurcating Level 0 to Level 3, and two, two, and one thermocouples were respectively decorated at each transverse channel. The remaining three thermocouples were located at another branch point for each bifurcating level for comparison. To provide a uniform heating condition, a foil heater with a heating area of 150 mm × 35 mm was tightly attached to the substrate bottom. The foil heater was connected to an AC power supply and could be regulated from 10 to 100 W. A detailed test section schematic is shown in Figure 4.

**Figure 5:**
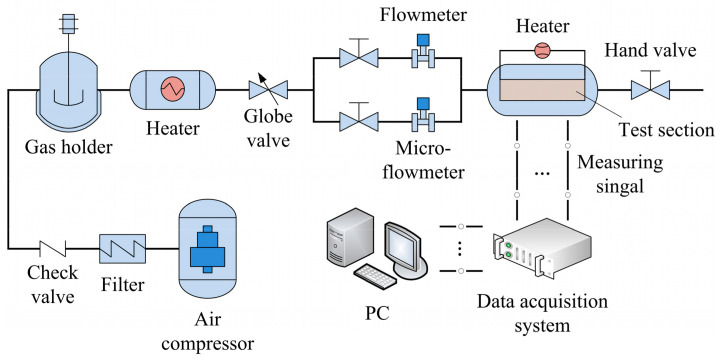
Schematic of the experimental apparatus.

Figure 5 presents a schematic diagram of the experimental apparatus for the heat transfer and pressure drop measurements for microchannels. This experimental rig consisted of an air compressor, a working section of the T-shaped bifurcating microchannel, a heating apparatus, a data acquisition, and a control system. First, the air coolant was drawn in through the dust filter and then passed into a gas holder for stabilizing pressure. The heater was then switched on for heating the air flowing through the branching microchannel with a certain wall heat flux. Finally, the air flow was exhausted into atmosphere. In order to minimize heat losses, all outside surfaces of the test section and foil heater as well as connecting pipes were thermally insulated with 50-mm-thick rubber insulation cotton.

For controlling the mass flow rate, a hand value was installed in front of the flowmeter, and the air flow rate was measured by a 0–25 L/min range metal float micro-flowmeter or a 20–200 L/min range vortex shedding flowmeter depending on the experimental conditions. The air pressure and temperature values at the channel inlet and outlet were measured with two pressure transducers and two type-K armored thermocouples with an outer diameter of 0.5 mm, respectively. A National Instruments (NI) data acquisition system (cDAQ-9178) was utilized for data reading and recordings. 

### 2.2. Data Reduction

Experimental results are rearranged into dimensionless representation. The local Nusselt number is defined by

(3) $$Nu_{k}=hd_{k}/\lambda$$

where *λ* is the air thermal conductivity, and *d_k_* is defined by the local hydraulic diameter of each bifurcation segment for the fractal-like network since such a network has no identical characteristic length. 

The heat transfer coefficient *h* is defined by

(4) $$h=Q/A\left(T_{w}-T_{b}\right)$$

where *A* denotes the total convection heat transfer area of the branching network microchannel.

The total heat removed by the cooling air is estimated by

(5) $$Q=mc_{p}\left(T_{\mathrm{out}}-T_{\mathrm{in}}\right)$$

where *m* is the air mass flow rate, *c_p_* is the air specific heat capacity, and *T*_out_ and *T*_in_ are respectively the air temperature at outlet and inlet, and read by the temperature transducers.

The location wall temperature *T*_w_ in Equation (4) is obtained from the thermocouple’s output. Supposing a linear temperature increase of air rises along the microchannel, the local bulk mean air temperature *T*_b_ is calculated by

(6) $$T_{b}=T_{\mathrm{in}}+\left(T_{\mathrm{out}}-T_{\mathrm{in}}\right)z/L.$$

The pressure drop Δ*p* across the microchannel test section is measured by the differential transducer. To obtain a non-dimensionalized pressure drop, the Fanning friction factor f is computed by

(7) $$f=\frac{\Delta p}{2\rho u_{\mathrm{in}}^{2}}\frac{d_{\mathrm{in}}}{L}$$

where *L* is the centerline length from inlet to outlet. *u*_in_ is the mean inlet velocity and calculated by utilizing the channel volume flow rate.

To appropriately estimate the overall thermal performance of the complex microchannel, the thermal enhancement factor for cooling air in the flowing channel is defined by

(8) $$\eta =\frac{Nu/Nu_{s}}{{\left(f/f_{s}\right)}^{1/3}}.$$

Here the Nusselt number for fully developed turbulent flow in a smooth microchannel is obtained from Stephan et al. [35] as

(9) $$Nu=4.364+\frac{0.086{\left(RePrd/L\right)}^{1.33}}{1+0.1Pr{\left(Red/L\right)}^{0.3}}.$$

The Filonenko correlation [36] for the friction factor in the smooth channel is

(10) $$f_{s}={\left(1.58\ln Re-3.28\right)}^{-2}.$$

To further evaluate the thermal performance of microchannels from the view of the second law of thermodynamics, the entropy generation rate caused by heat exchange between the coolant and the heating wall can be expressed by [37]

(11) $${\dot{S}}_{t}=\frac{Q}{T_{b}}-\frac{Q}{T_{heating}}=\frac{Q\left(T_{heating}-T_{b}\right)}{T_{b}T_{heating}}$$

where *T_heating_* is the average temperature of the heating wall.

Then, the entropy generation rate induced by the flow friction is defined by

(12) $${\dot{S}}_{p}=-m\left(\int_{p_{in}}^{p_{out}}\frac{1}{\rho T_{b}}dp\right)=\frac{m\Delta p}{\rho T_{b}}.$$

Further, by combining Equations (11) and (12), the total entropy generation rate in the flow field can be arranged to give

(13) $${\dot{S}}_{gen}={\dot{S}}_{t}+{\dot{S}}_{p}=\frac{Q\left(T_{heating}-T_{b}\right)}{T_{b}T_{heating}}+\frac{m\Delta p}{\rho T_{b}}.$$

To quantify the total entropy generation rate, the augmentation entropy generation number is defined by

(14) $$N_{s}={\dot{S}}_{gen}/{\dot{S}}_{gen,s}.$$

where ${\dot{S}}_{gen,s}$ is the total entropy generation rate through the smooth branching microchannel.

The experimental uncertainties are determined by a standard uncertainty analysis approach [38]. The uncertainty (at the 95% confidence level) is ±13.5% for the Nusselt number and ±8.5% for the flow friction factor, respectively.

## 3. Numerical Method

**Figure 6:**
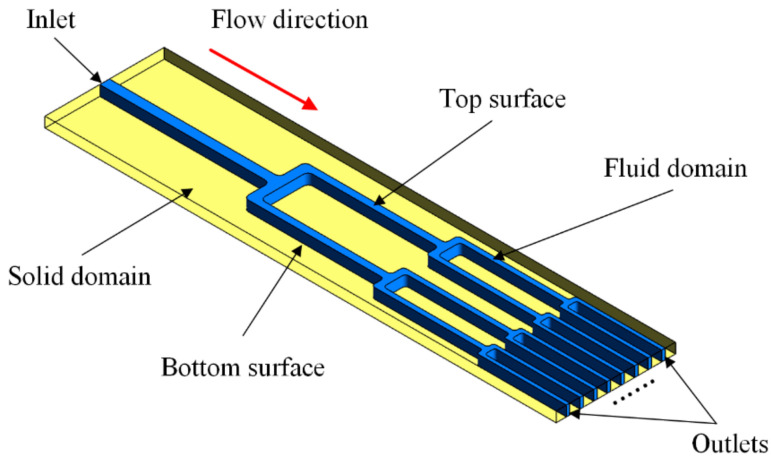
Schematic of the boundary conditions for the branching microchannel calculation.

**Figure 7:**
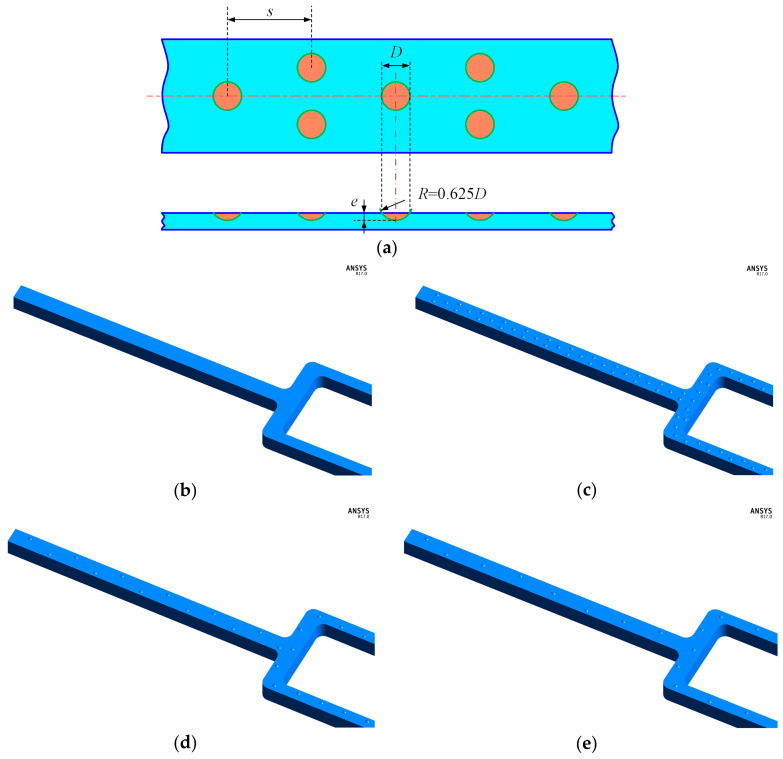
Geometric model: (**a**) Geometrical parameters of the dimple; (**b**) non-dimples; (**c**) dimples with *s*/*D* = 3; (**d**) dimples with *s*/*D* = 6; and (**e**) dimples with *s*/*D* = 9.

To gain comprehensive physical understandings on the detailed hydrodynamics and thermodynamic characteristics in the tree-like branching microchannel with and without dimples, the three-dimensional steady-state numerical computations were implemented. Figure 6 presents a schematic of the boundary conditions for the calculations on the heat transfer and flow in the branching microchannel, and the boundary conditions are consistent with those in the experimental work. In the calculation, a static temperature of 300 K is imposed at the inlet of the solution domain, a static backpressure is set at the outlet domain, and the thermal boundary condition with a constant wall heat flux of 6000 W/m^2^ is applied at the bottom surface. The *Re* number is imposed at the inlet, and the value varies so that it is consistent with the experiment. We also examined three different dimple arrangement patterns. The branching microchannel with dimples were then compared to the microchannel without dimples. The structures depicting these three dimples cases are demonstrated in Figure 7. The spherical dimple has a print diameter *D* = 0.7 mm with a depth of 0.14 mm (depth to diameter *e*/*D* = 0.2), and streamwise spacing *s* = 2.1 mm, 4.2 mm, and 6.3 mm (streamwise spacing to diameter *s*/*D* = 3, 6 and 9). The fractions of the top surface covered by the dimples are respectively about 14.0%, 6.7%, and 4.7%.

The numerical simulations for the hydrodynamics and thermodynamic in tree-like branching microchannels were conducted using the commercial CFD software package ANSYS CFX V17.0 Assuming an incompressible and steady flow, the continuity, momentum, and energy governing equations were solved by the fully implicit approach. Based on a comparison among the standard *k*-*ε* turbulence model, the RNG *k*-*ε* turbulence model, and the SSG turbulence model for the heat transfer and flow pressure drop predictions in the smooth branching channel (shown below), the SSG turbulence model was chosen for the calculations. In a report on ANSYS CFX software [39], the SSG model provides good predictions of the features and physics of most flows, and is suitable for the secondary flow and the flow with strong streamline curvature or sudden changes in the mean strain rate. Thereby, the SSG model is recommended for the complex air flow in the branching microchannels with dimples. The near-wall region is managed by the scalable wall function, which can well solve the problem of contradictions in the wall function in the context of fine meshes.

**Figure 8:**
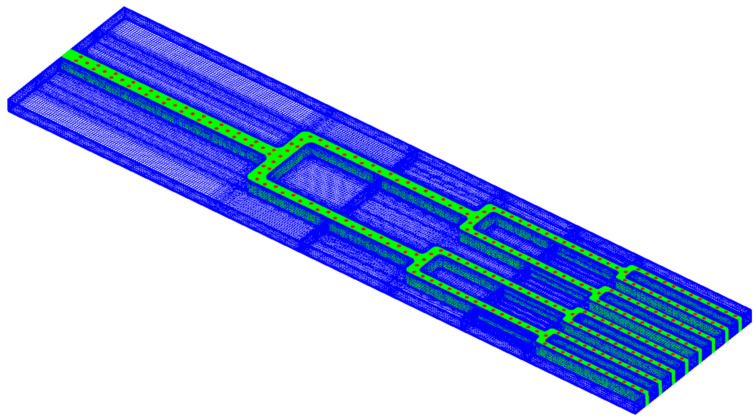
Mesh in computational domain (dimples case with *s*/*D* = 3).

As presented in Figure 8, the computational domain mesh is discretized to fine cells to implement the simulation. A local mesh refinement is employed in the near-wall flow region, and *y* plus value of the first cell is limited to 1 in all cases. To verify the accuracy of the computation, the grid independence of four structures was verified. For the case without dimples, the appropriate mesh size falls in the range of 9.72 million cells. For the other three structures with dimples, the appropriate mesh numbers were set to 19.98 million, 18.93 million, and 18.69 million, respectively.

Table 2 and Table 3 present the results of three turbulence models for the heat transfer and flow pressure drop in the branching microchannel without dimples. It can be seen that the SSG turbulence model overpredicts the heat transfer in the branching microchannel, with reasonable deviations less than 26%, and predicts the flow friction factor with acceptable deviations of about 6.5%. The standard *k*-*ε* turbulence model and RNG *k*-*ε* overpredict the averaged *Nu* numbers with a deviation of about 47.4% and 38.2%, but well predict the friction factors, with deviations of about 0.8% and 9.4%. Considering the heat transfer and flow performance overall, the SSG turbulence model was chosen for all calculations.

## 4. Results and Discussions

### 4.1. Experiment Results and Numerical Verification

**Figure 9:**
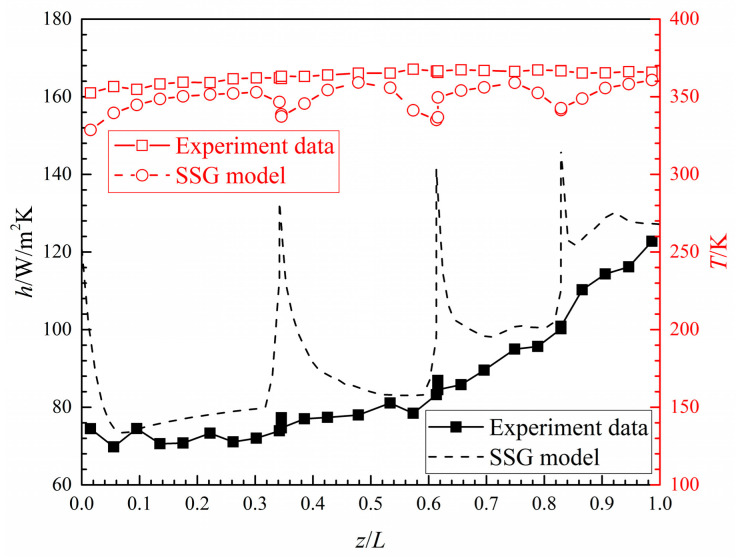
Experimental results and numerical data for the local heat transfer coefficient and wall temperature of the smooth microchannel.

**Figure 10:**
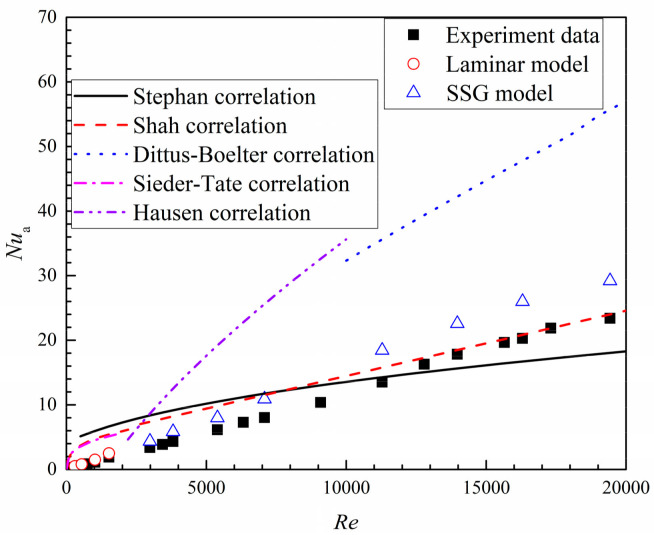
Experimental results and numerical data as well as the correlations for averaged *Nu* numbers of the smooth microchannel.

Figure 9 illustrates the experimental heat transfer coefficient and wall temperature distribution of the smooth branching microchannel, which are shown with the numerical results in the case of *Re* = 5400. It is apparent that the changing trend of the numerical data is in agreement with the experimental results, though showing a large difference at each bifurcation. Figure 10 presents the experimental results of the averaged *Nu* numbers of the smooth tree-like branching microchannel. Over the studied *Re* numbers from 200 to 20,000, the smooth microchannel shows a *Nu* value from 0.2 to 24. The experimental data was compared with the correlations of Stephan [35], Shah [40], Dittus-Boelter, Sieder-Tate [41], and Hausen [42] and is lower than all correlations within the *Re* number of 200–10,000. After that, with increasing *Re* numbers, the experimental results are in good agreement with the correlation from Shah. Figure 10 also shows that, compared to the experimental data, the numerical results of the averaged *Nu* numbers are overestimated by about 26.7% within the investigated *Re* number. The large deviations between the computations and experiment are due to the influences of the large wall thermal conduction and the strong conjugate heat transfer effects in the micro-scale channel. These effects, which not only inhibit the temperature fluctuation in each bifurcation but also depress the averaged heat transfer level, have been discussed in the literature [34].

**Figure 11:**
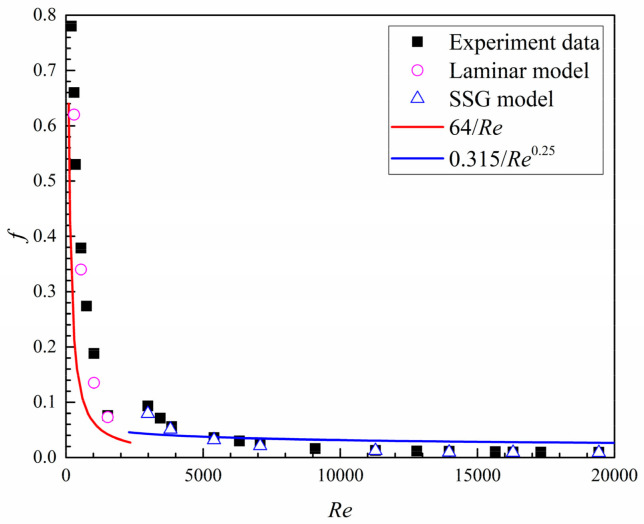
Experimental results and numerical data as well as the correlations for the friction factor of the smooth microchannel.

Figure 11 shows the experimental and numerical data of the flow friction factors of the tree-like branching microchannel without dimples. Compared to the experimental results, the numerical data is underestimated by about 29.2% in the laminar flow region, but they are close with each other in the turbulent flow region. In the figure, the classical correlations of *f* = 64/*Re* for *Re* < 2300 and *f* = 0.315/*Re*^0.25^ for *Re* > 2300 are also plotted. Both the experimental and numerical data are above the classical correlation curve in the laminar flow region but below it in the turbulent flow region. These comparative results suggest that the diffuser effect of the branching structure contributes to a marked pressure recovery in the turbulent region and thus decreases the flow friction loss.

### 4.2. Comparison of Flow and Heat Transfer Characteristics between Smooth and Dimpled Branching Microchannel

**Figure 12:**
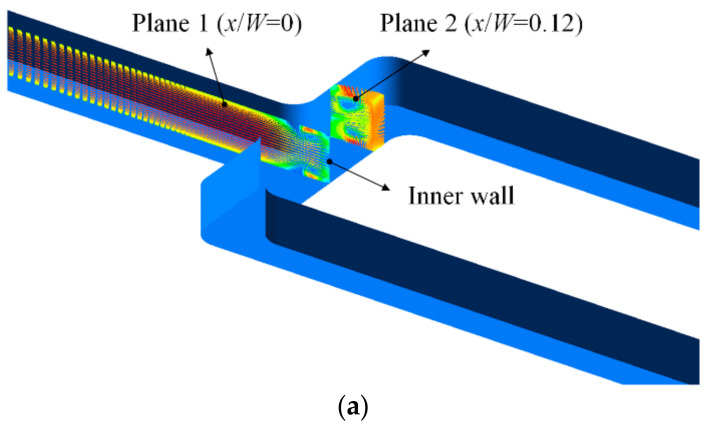

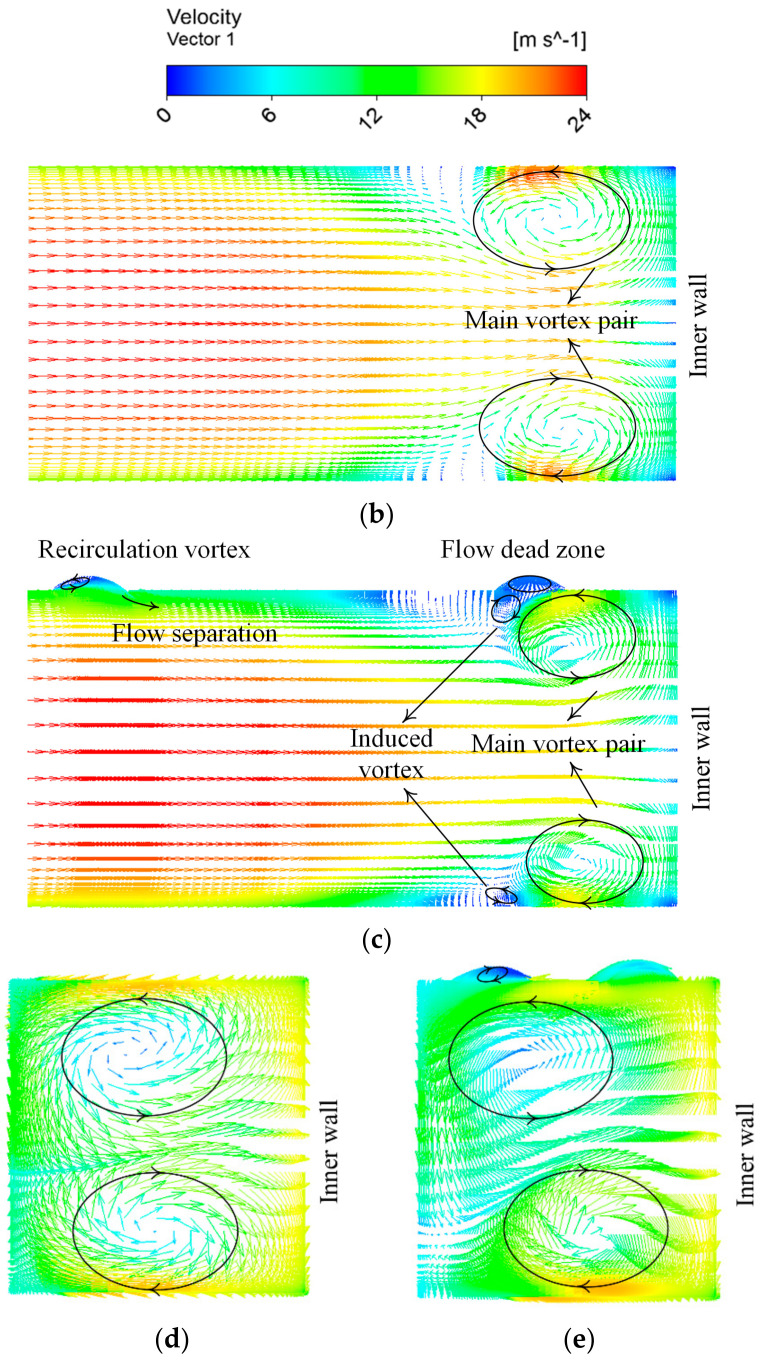
Velocity vector distribution on the symmetry plane at *Re* = 5400: (**a**) location of the cut planes; (**b**) plane1 in the smooth branching microchannel; (**c**) plance1 in the dimpled branching microchannel; (**d**) plane2 in the smooth branching microchannel; (**e**) plane2 in the dimpled branching microchannel.

Figure 12 shows the detailed velocity vector distribution on the cut planes at *Re* = 5400. On the plane 1 (*x*/*W* = 0) of the smooth microchannel, it is clear that the T-shaped branching structure causes a strong impinging jet flow near the inner wall and then forms a pair of secondary flow vortexes. The main vortex motion markedly promotes the convective heat transfer process. On the same plane of the dimpled microchannel, the scale of vortex pair generated by the branching structure is reduced. Moreover, between the primary vortex ring and the wall, a pair of small induced vortexes is generated. Moreover, the dimple produces the recirculation flow vortex in the upstream half of the dimple and flow separation in the adjacent downstream area, which significantly perturbs the flow motion near the wall region and thus further enhances the heat transfer. However, the flow velocity in the last dimple near the inner wall is very slow and almost appears stagnant. The existence of the flow dead zone impedes the fluid flow and suppresses the heat transfer. With the coolant flow moving downstream, the flow velocity vector on Plane 2 (*x*/*W* = 0.12) of the smooth microchannel still exhibits a symmetrical flow pattern, whereas the vortex core locations of the secondary vortex pair in the dimpled microchannel change, resulting in fluid flow acceleration near the wall region.

**Figure 13:**
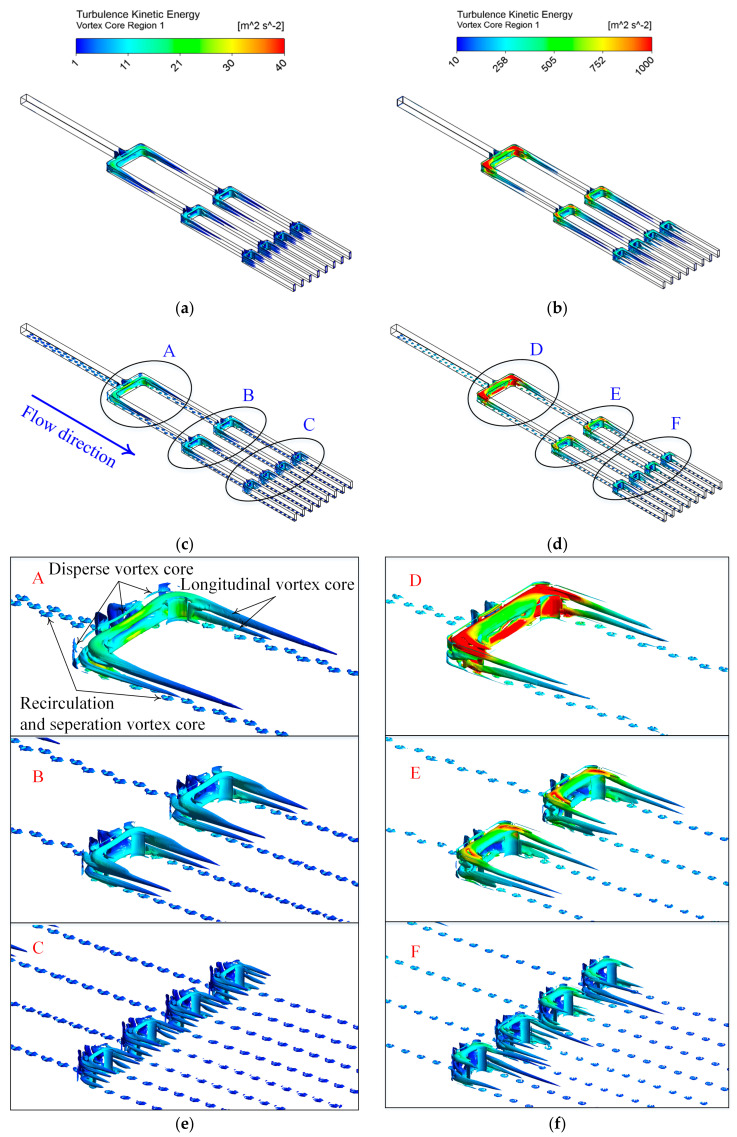
Contour plot of turbulence kinetic energy on the vortex core region of the branching microchannels with and without dimples at two different *Re* numbers, respectively. The combination of different parameters chosen for the plotting are as follows: (**a**) *Re* = 5400 in the smooth branching microchannel; (**b**) *Re* = 50,000 in the smooth branching microchannel; (**c**) *Re* = 5400 in the dimpled branching microchannel; (**d**) *Re* = 50,000 in the dimpled branching microchannel. The partially enlarged view of the dimpled branching microchannel for two *Re* numbers are (**e**) *Re* = 5400 and (**f**) *Re* = 50,000.

For a more in-depth analysis of the gross characteristics of the flow field and its effect on local *Nu* number distribution, the distribution law of vortex cores in the branching microchannel with and without dimples at *Re* = 5400 and 50,000 are shown in Figure 13. The vortex core characterized by the secondary flow in the channel mainly consist of the longitudinal secondary flow induced by branches, as well as the recirculation and separation secondary flow caused by dimples. The secondary flow field can be identified by several methods, such as the *Q*-criterion, the helicity criterion, the swirling criterion, the vorticity criterion, and the *λ*_2_ criterion. The *λ*_2_ criterion can provide an accuracy that is satisfactory for describing the secondary flow field, and this has been proven by Jeong and Hussain [43], so it is adopted to discuss the vortex core on heat transfer of the channel in the present investigation. The contour on the vortex core surface represents the contour of turbulence kinetic energy. In the figure, as the *Re* number changes from 5400 to 50,000, the development of the separation flow vortex region downstream of the dimples is significantly suppressed, but the structure of the longitudinal and disperse vortex core regions in the dimpled branching microchannel displays no obvious variation, while the scale of the longitudinal vortex core region in the smooth branching microchannel becomes significantly longer. Generally, the strength of the turbulence intensity will have a strong impact on convective heat transfer enhancement. Moreover, with the increase in the *Re* number, the vortex regions of branching microchannels with and without dimples both show the strengthening in the turbulence kinetic energy level. Under the same *Re* number, however, the length of the primary longitudinal vortex core after the bend is shorter in the dimpled channel than that in the smooth one. The compared result implies that the presence of dimples restrains the development of the longitudinal vortex along the flow direction.

**Figure 14:**
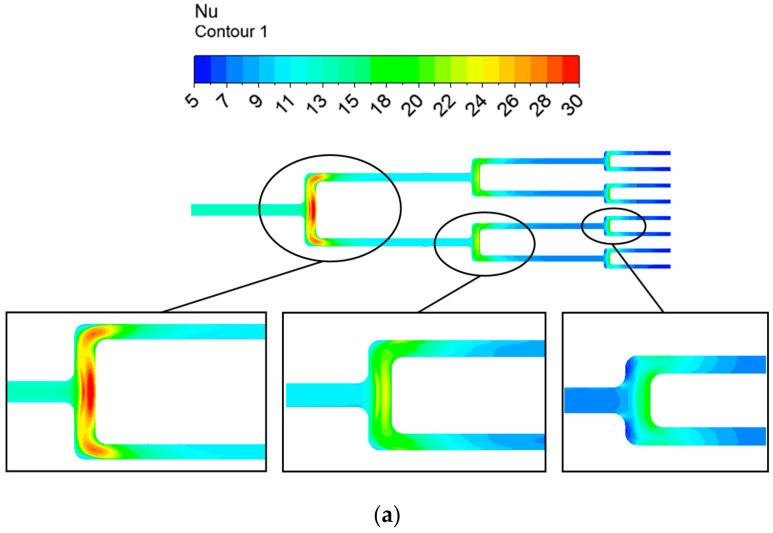

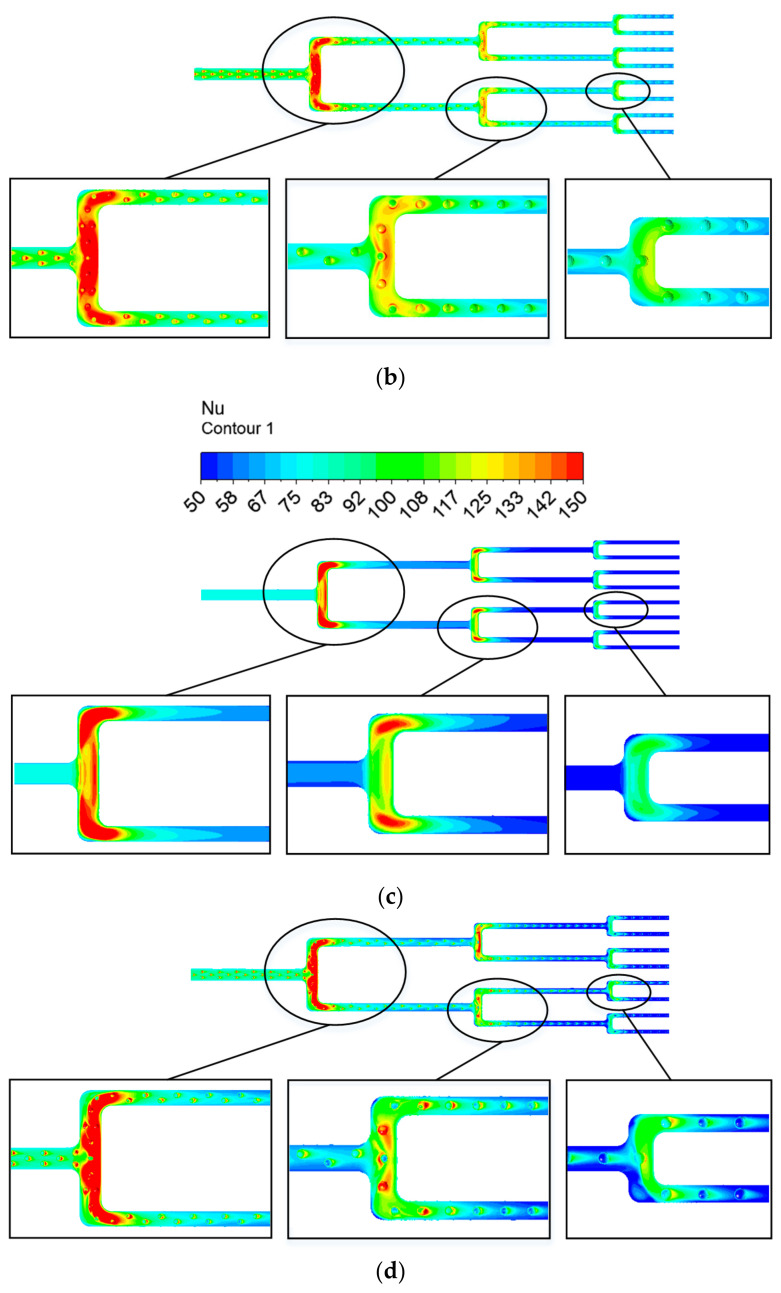
Contour plot of *Nu* numbers on the top wall: (**a**) *Re* = 5400 with a smooth surface; (**b**) *Re* = 5400 with a dimpled surface; (**c**) *Re* = 50,000 with a smooth surface; (**d**) *Re* = 50,000 with a dimpled surface.

Figure 14 displays the contour plots of the *Nu* number distribution on the smooth and dimpled surfaces at *Re* = 5400 and 50,000. As shown in the figure, the distribution laws of local *Nu* numbers at two *Re* numbers are similar. It can be seen that high heat transfer regions always locate relevant to the high strength vortex core regions, so the highest heat transfer level is found at each branch where the longitudinal vortex displays the strongest turbulence kinetic energy. Compared to the smooth surface, the presence of recirculation flow in the dimples and the separation vortexes on the flat surface downstream of dimples enhances the overall heat transfer performance in the entire channel, although the flow dead zones in some dimples at bifurcations and bends inhibit the heat transfer of coolant. Furthermore, with an increased branching level, the channel diameter is reduced, so the secondary flow disturbance induced by dimples is more violent, accordingly further promoting the heat transfer.

**Figure 15:**
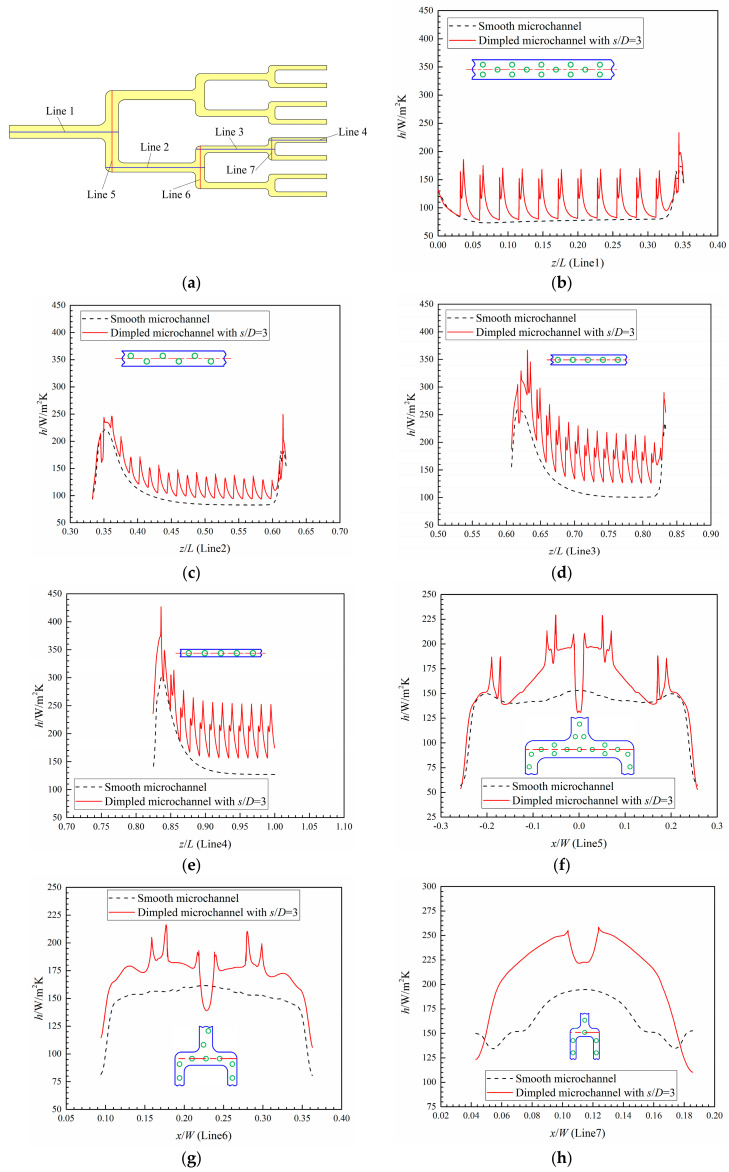
(**a**) Detailed line locations in the branching microchannel and the local heat transfer coefficient distribution in (**b**) Line1, (**c**) Line2, (**d**) Line3, (**e**) Line4, (**f**) Line5, (**g**) Line6 and (**h**) Line7.

Figure 15 compares the numerical results of the local heat transfer coefficient along the centerlines of top surfaces for each branch segment from Bifurcating Level 0 to Level 3 at *Re* = 5400. The detailed location of each centerline is shown in Figure 15a. Along the *z* direction (see Figure 15b–e), it can be found that the local heat transfer level near the dimple regions changes significantly and periodically, and the dimple surface displays a higher averaged heat transfer coefficient than that of the counterpart of the smooth surface for each branch segment by about 26.5%, 14.3%, 25.0%, and 32.6%, respectively.

Along the *x* direction, the variations of the local heat transfer level in the transverse microchannel are presented in Figure 15f–h. In the figure, it can be seen that the averaged heat transfer coefficient over the dimpled surface is, respectively, about 12.0%, 17.9%, and 32.4% higher than that of the counterpart of the smooth surface for each branch segment. It should be noted that, compared to the smooth transverse microchannel, the heat transfer characteristics of the dimpled transverse microchannel becomes quite complex due to the mixing effects of fluid diffusion, recirculation, and separation motions. 

### 4.3. Thermal Enhancement Performance of Dimpled Branching Microchannels

**Figure 16:**
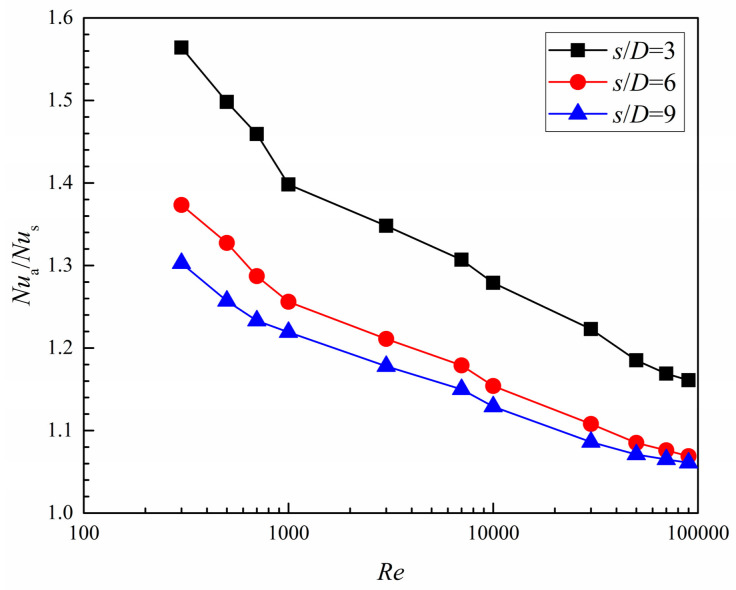
Averaged *Nu* number ratio of the dimpled branching microchannel.

It is desirable to analyze the heat transfer performance of the dimpled branching microchannel, but it is also interesting to quantify the degree of the heat transfer augmentation relative to the corresponding smooth microchannel for different dimple arrangement patterns. The variations of the averaged *Nu* number ratio (*Nu*_a_/*Nu*_s_), on the dimpled side walls for the streamwise spacing to diameter of *s*/*D* = 3, 6, and 9 are shown in Figure 16. The *Nu*_a_/*Nu*_s_ appears to decrease as *Re* number increases. The decline rate is greater for *Re* numbers from 300 to 1000 (in the laminar flow region) and decreases for *Re* = 3000–90,000 (in the turbulent flow region). The maximum *Nu*_a_/*Nu*_s_ is acquired for *s*/*D* = 3, while the lowest one is for *s*/*D* = 9. The increases in *Nu*_a_/*Nu*_s_ values for conducting *s*/*D* = 3, 6, and 9 are respectively about 1.56, 1.37, and 1.30 at the lowest *Re* number, and 1.16, 1.07, and 1.06 at the highest *Re* number.

**Figure 17:**
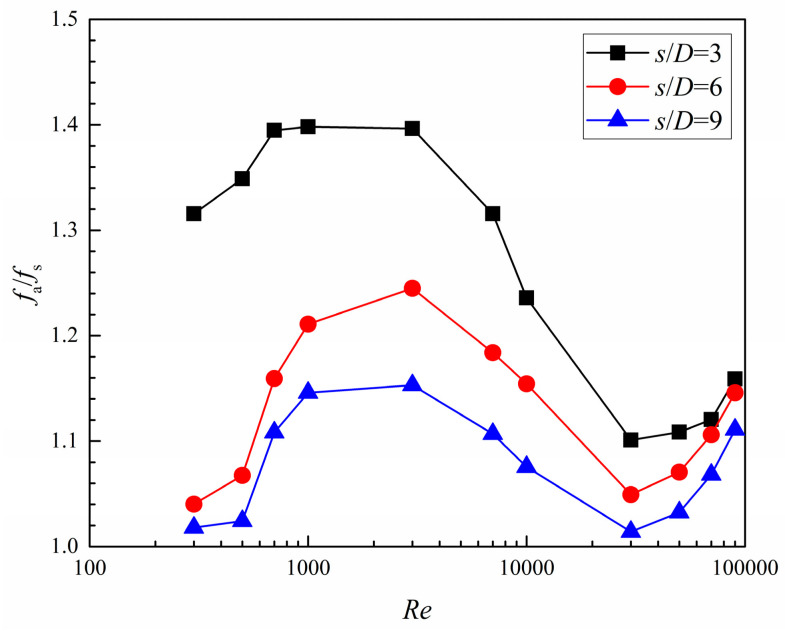
Friction factor ratio of the dimpled branching microchannel.

Because the heat transfer enhancement in the dimpled channel is typically accompanied by an augmentation in pressure loss, a synthetic study of performance should include the influence of dimpled surfaces on the channel pressure loss. The friction factor ratio (*fa*/*f*_s_) in the same *s*/*D* cases depicted in Figure 17 rises first with the increase in *Re* number and obtains a peak value at *Re* = 3000, and subsequently drops rapidly a valley value at *Re* = 30,000. Afterward, the value rises gradually. The more densely populated dimples generate a more intense turbulent flow, resulting in a greater pressure loss. The *fa*/*f*_s_ increment of the dimpled branching microchannel with *s*/*D* = 3, 6, and 9 can be respectively 1.10–1.40, 1.05–1.24, and 1.01–1.15 times the fully developed flow in the corresponding smooth branching microchannel.

**Figure 18:**
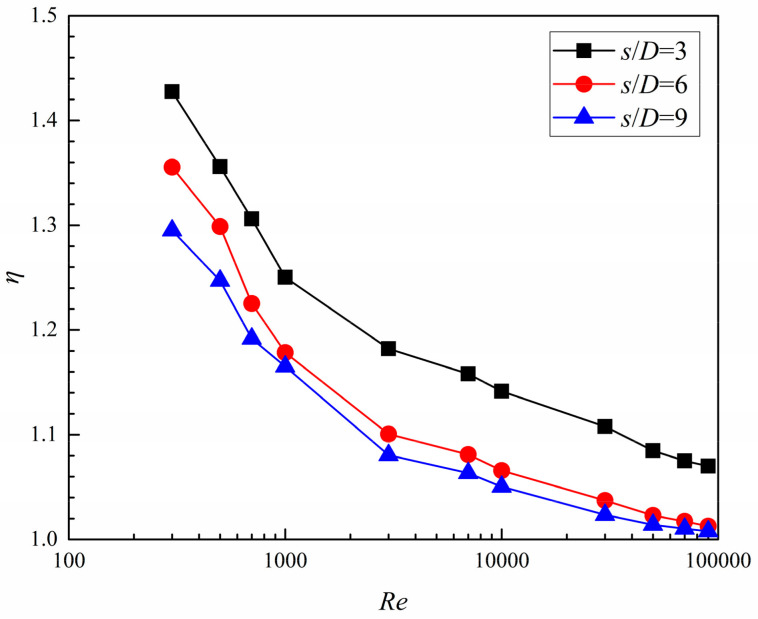
Thermal enhancement factor of the dimpled branching microchannel.

With the averaged *Nu* number increment and flow friction factor increment, the thermal enhancement factor (*η*) for the dimpled branching microchannel can be evaluated. Figure 18 presents the variation of *η* for the branching microchannel with different dimple arrangement patterns. The *η* tends to decrease as the *Re* number increases in the same dimple arrangement. The case of *s*/*D* = 3 obtains a higher *η* than the other cases, with the value varying from 1.42 to 1.07. As the *s*/*D* increases from 6 to 9, the *η* slightly declines.

**Figure 19:**
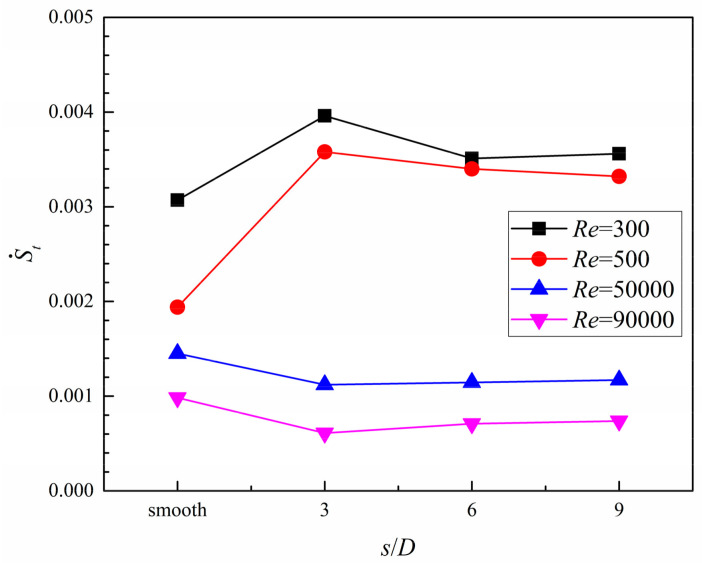
The effects of dimple arrangement pattern on entropy generation induced from heat transfer.

**Figure 20:**
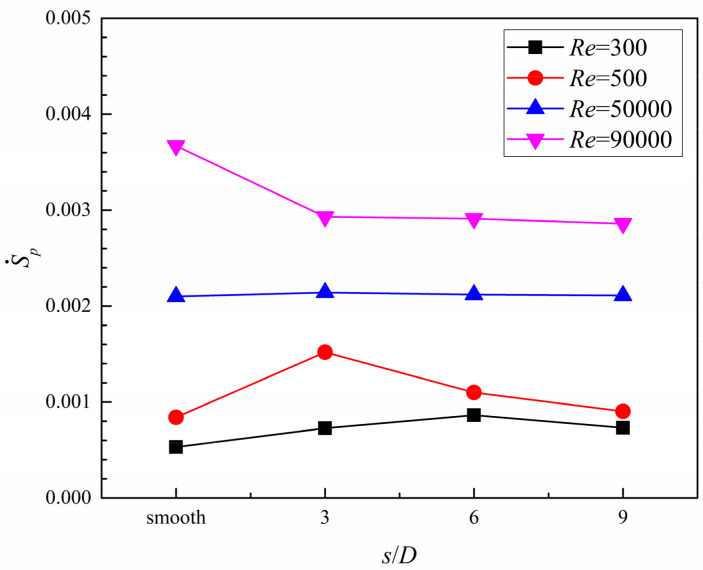
The effects of dimple arrangement pattern on entropy generation induced from fluid friction.

Entropy generation and an optimal dimple arrangement pattern under different *Re* number conditions, including *s*/*D* = 3, 6, and 9, are exemplified in Figure 19 and Figure 20. A clear trend can be seen in Figure 19: In the laminar flow region (*Re* = 300 and 500), ${\dot{S}}_{t}$ in all dimpled cases is larger than that in the case without dimples. In the turbulent flow region (*Re* = 50,000 and 90,000), the compared results show the contrary. Moreover, the value of ${\dot{S}}_{t}$ in all cases are decreased as the *Re* number rises, suggesting that the heat exchange capacity of the coolant is enhanced; thus, the irreversibility loss caused by the unbalanced temperature difference between coolant and heating surface is decreased with the increase in *Re* number.

As shown in Figure 20, under a low *Re* number condition, ${\dot{S}}_{p}$, in the smooth case, is firstly higher than that in all dimpled cases and gradually drops as the *Re* number increases. Furthermore, the value of ${\dot{S}}_{p}$ in all cases increases as the *Re* number increases, meaning the irreversibility loss induced by the fluid flow friction, namely the pressure drop through the microchannel is increased as the *Re* number increases.

**Figure 21:**
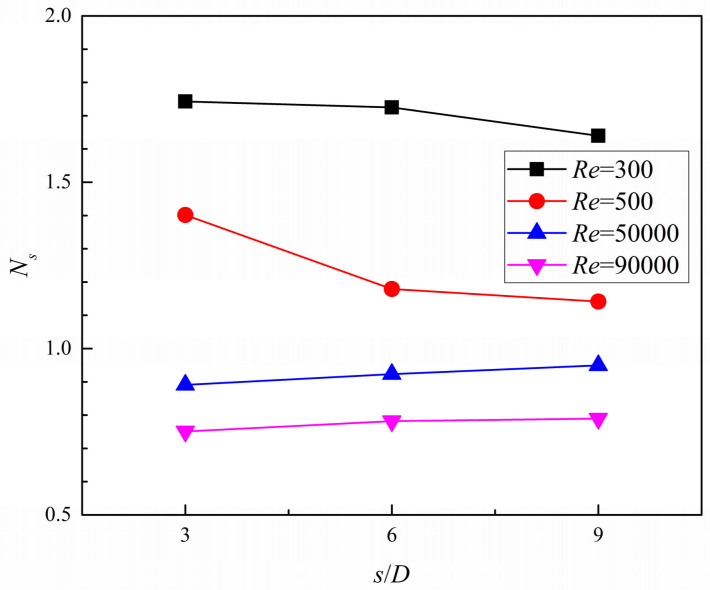
Augmentation entropy generation number of the dimpled branching microchannel.

The effect of the *Re* number on the augmentation entropy generation number (*N_s_*) with different dimple arrangement pattern is depicted in Figure 21. The *N_s_* changes as a function of the *Re* number and when the value of *N_s_* is well below unity, the microchannel will obtain the best performance. In the figure, it is clear that the ratio of *N_s_* reduces as the *Re* number increases. When the coolant flows in the turbulent flow region, the value of *N_s_* shows lower than unity. This indicates that the irreversibility loss of dimpled cases is decreased compared with that of the smooth case under high *Re* number conditions. Moreover, the value of *N_s_* in the dimple case with *s*/*D* = 3 is lower than that in the other two dimpled cases under high *Re* number conditions (*Re* = 50,000 and 90,000), while an obvious contrast is shown under low *Re* number conditions (*Re* = 300 and 500).

## 5. Conclusions

A new internal cooling structure of the tree-like branching microchannel combined with dimples orientated to gas turbine blade was designed. The flow structure and heat transfer performance were investigated numerically. The results of verification experiments for the smooth branching microchannel show that the SSG turbulence model can provide a sufficient prediction accuracy of the flow and heat transfer details.

The three-dimensional numerical simulations reveal that the dimples produce recirculation and flow separation, and change the vortex core locations of the secondary vortex pair leading to the fluid flow acceleration in the near-wall flow region, thus generating superior local and overall hat transfer augmentation. Compared to the smooth microchannel, the branch effects are more obvious with respect to the heat transfer development in the dimpled microchannel. The higher the branching level is, the greater the heat transfer increment of the dimpled branching microchannel becomes.

Results of comparison with three dimple arrangement patterns in the branching microchannel show that, as *Re* number increases from 300 to 90,000, *Nu*_a_/*Nu*_s_ and *η* monotonously decrease; however, *f*_a_/*f*_s_ rises to *Re* = 3000, falls to *Re* = 30,000, and then gradually increases afterward. Moreover, *Nu*_a_/*Nu*_s_, *f*_a_/*f*_s_, and *η* are decreased when *s*/*D* increases from 3 to 9.

Based on the analysis of entropy generation, it was found that, with the increase in the *Re* number, the values of ${\dot{S}}_{t}$ decreased and those of ${\dot{S}}_{p}$ increased in all cases. The dimpled cases, compared with the smooth case under high *Re* number conditions, show lower entropy generation rates induced from heat transfer and fluid flow friction. Furthermore, when the coolant flow in the turbulent flow region, the ratio of *N_s_* of all dimpled cases are lower than unity, and the dimpled case with *s*/*D* = 3 gains the lowest value. From the view of comprehensive heat transfer augmentation and energy savings, the dimpled branching microchannel with *s*/*D* = 3 exhibits the best performance.

## Figures and Tables

**Table 1:** The geometric parameters of the tree-like branching microchannel.

*k*	*l_k_* (mm)	*d_k_* (mm)	*H_k_* (mm)	*W_k_* (mm)	*S_k_* (mm)
0	51.29	2.91	3	2.83	-
1	40.72	2.06	3	1.57	18.0
2	32.32	1.46	3	0.96	9.4
3	25.65	1.03	3	0.62	5.0

**Table 2:** Turbulence models for heat transfer prediction in the branching microchannel without dimples.

*Nu*	CFD Data (Standard *k*-*ε* Model)	CFD Data (RNG *k*-*ε* Model)	CFD Data (SSG Model)	Experimental Data
*Re* = 5400	9.6	8.8	7.9	6.3
*Re* = 11300	20.8	20.0	18.4	14.6

**Table 3:** Turbulence models for flow friction factor prediction in the branching microchannel without dimples.

*f*	CFD Data (Standard *k*-*ε* Model)	CFD Data (RNG *k*-*ε* Model)	CFD Data (SSG Model)	Experimental Data
*Re* = 5400	0.0360	0.0305	0.032	0.0359
*Re* = 11300	0.0131	0.0124	0.0125	0.0129
